# Digital Intervention (Keep-On-Keep-Up Nutrition) to Improve Nutrition in Older Adults: Protocol for a Feasibility Randomized Controlled Trial

**DOI:** 10.2196/50922

**Published:** 2024-04-30

**Authors:** Chloe French, Sorrel Burden, Emma Stanmore

**Affiliations:** 1 School of Health Sciences University of Manchester Manchester United Kingdom

**Keywords:** feasibility, usability, digital health, diet, gerontology, geriatric, geriatrics, older adult, older adults, elder, elderly, older person, older people, ageing, aging, dietary, nutrition, hydration, community dwelling, RCT, randomized, controlled trial, controlled trials

## Abstract

**Background:**

Digital health tools can support behavior change and allow interventions to be scalable at a minimal cost. Keep-on-Keep-up Nutrition (KOKU-Nut) is a free, tablet-based app that focuses on increasing physical activity and improving the dietary intake of older adults based on UK guidelines. The intervention targets an important research area identified as a research priority reported by the James Lind Alliance priority setting partnership for malnutrition.

**Objective:**

This study aims to assess the feasibility of using the digital health tool KOKU-Nut among community-dwelling older adults to inform a future randomized controlled trial. The secondary aims are to determine the acceptability, usability, preliminary effect sizes, and safety of the study and the intervention (KOKU-Nut).

**Methods:**

This is a feasibility randomized controlled trial. We plan to recruit a total of 36 community-dwelling older adults using purposive sampling. Participants will be randomized 1:1 to either the intervention or the control group. The intervention group will be asked to engage with KOKU-Nut 3 times a week for 12 weeks. Participants in the control group will receive a leaflet promoting a healthy lifestyle. All study participants will complete questionnaires at baseline and the end of the 12 weeks. A sample of participants will be asked to participate in an optional interview. The study will collect a range of data including anthropometry (height and weight), dietary intake (3-day food diary), physical function (grip strength and 5-times sit-to-stand), perceived quality of life (EQ-5D), usability (System Usability Scale), and safety (adverse events).

**Results:**

Data collection commenced in March 2024, and the results will be ready for publication by January 2025. Feasibility will be determined on the basis of participants’ self-reported engagement with the intervention, and recruitment and retention rates and will be summarized descriptively. We will also consider the amount of missing data and assess how outcomes are related to group assignment. Acceptability will be measured using the modified treatment evaluation inventory and one-to-one semistructured interviews. Transcripts from the interviews will be analyzed using NVivo (version 12; QSR International) software using framework analysis to understand any barriers to the recruitment process, the suitability of the assessment measures, and the acceptability of the intervention and study design.

**Conclusions:**

The study aligns with guidelines developed by the Medical Research Council for developing a complex intervention by using qualitative and quantitative research to examine the barriers of the intervention and identify potential challenges around recruitment and retention. We anticipate that these results will inform the development of a future powered randomized controlled design trial to test the true effectiveness of KOKU-Nut.

**Trial Registration:**

ClinicalTrials.gov NCT05943366; https://classic.clinicaltrials.gov/ct2/show/NCT05943366

**International Registered Report Identifier (IRRID):**

PRR1-10.2196/50922

## Introduction

It is well established that progressive resistance training combined with strength and balance exercises are safe and effective for improving muscle mass, hip and lumbar spine bone mineral density, and muscle strength and function [1] and for reducing the risk of falls [2], hospitalizations, fractures [3], and mortality. Furthermore, diet has a fundamental role in the health of older adults [4], and meta-analyses have found that nutritional interventions in addition to physical activity lead to greater improvements in physical function, body composition, and strength in frail and pre-frail older adults [5-7].

Advancements in technology have provided the opportunity for digital platforms to remotely deliver, monitor, and complement patient care. Digital platforms can complement traditional face-to-face programs or provide an alternative option based on the individual’s preference and financial situation [8]. Furthermore, digital interventions can be scalable so they can maximize outreach at a minimum cost. Mobile apps are increasingly being used to support dietary change [9] and increase physical activity [10]; however, most are not designed for the older adult population. A recent systematic review identified that apps can be effective at increasing physical activity levels in community-dwelling older adults [8]; however, there has been little research investigating the effectiveness of apps to prevent malnutrition in this population [11].

Keep-on-Keep-up (KOKU) is a free, tablet-based gamified strength and balance exercise app that was soft launched in 2020 [12]. KOKU is approved by the National Health Service and Organization for the Review of Care and Health Apps as a lifestyle app and has been viewed positively by older adults after 6 weeks of independent use [13]. Anonymized Google analytics data show that there are more than 1000 users in the United Kingdom and users averaged 2.2 sessions per week, with an average weekly engagement of 1 hour 51 minutes. KOKU has a collection of health literacy games, and studies internationally found that engagement with the app led to increased exercise frequency and qualitative feedback found that the gamification was engaging and motivating [13]. Keep-on-Keep-up-Nutrition (KOKU-Nut) is a development of this gamification platform and includes an educational and interactive game based on the UK dietary guidelines to nudge older adults to improve their diet [14,15]. The game requires the user to choose a pair of cards to reveal food items, with the aim of finding matching food items. The user is presented with information about the different food items, and upon finding all the matching pairs, the user is provided with important tips to improve their diet and reduce their risk of malnutrition.

The intervention is based on qualitative work with 33 older adults to understand their determinants of dietary intake and barriers to healthy eating. Initial prototypes were tested on a diverse group of end users and health care professionals to facilitate co-design and ensure that the needs of the user were taken into consideration.

This study aims to assess the feasibility of conducting a randomized controlled trial (RCT) using KOKU-Nut to improve the dietary intake of community-dwelling older adults. The secondary aims are to determine the acceptability, usability, preliminary effect sizes, and safety of the study and the intervention (KOKU-Nut). These aims are in line with the Medical Research Council Framework that advocates conducting a feasibility study as part of the development and evaluation of complex interventions to assess the study design and the intervention itself [16].

## Methods

### Study Setting

This is a feasibility parallel group RCT study design using 1:1 randomization using both quantitative and qualitative methods to explore the feasibility and acceptability of KOKU-Nut (a digital service that aims to improve nutritional and fluid intake in community-dwelling older adults). Data will be collected from community-dwelling older adults living in Greater Manchester in the United Kingdom. The SPIRIT (Standard Protocol Items: Recommendations for Interventional Trials) statement and CONSORT (Consolidated Standards of Reporting Trials) statement extended to randomized pilot and feasibility trials and associated checklist were used in the design and reporting of this study [17,18] (Multimedia Appendix 1). Version 3 of the protocol was last updated on February 5, 2024.

### Eligibility Criteria

Participants will be included in the study if they are aged 65 years or older, living independently in the community, have access to the internet, and are willing to use an iPad or tablet (their own or one provided) for the duration of the study. Participants will be excluded if they are unable to communicate in English or have a known cognitive impairment.

### Interventions

Participants in the intervention group will be helped to download KOKU-Nut onto their iPad or tablet during the baseline visit. The researcher (CF) will demonstrate the features and assist with any technical queries. In cases where participants do not have the necessary devices or data to join the intervention, a tablet with KOKU-Nut installed will be provided for the duration of the intervention. Participants will be asked to engage with KOKU-Nut at least 3 times a week throughout the 12-week period. This will involve carrying out strength and balance exercises and engaging with the gamification features relating to bone health and healthy eating. A crib sheet and contact details for the research team will be available if participants require additional support to help with technical issues.

Participants assigned to the control group will continue with usual care and receive a leaflet developed by Age UK about the importance of a healthy lifestyle including information on the importance of staying active and nutrition [19]. This will allow the comparison of a more traditional way to provide lifestyle advice compared to the digital health tool.

### Outcomes

Primary outcomes will be related to feasibility and will assess engagement with the intervention, recruitment and retention rates, and acceptability of the intervention and study design. These findings are important to inform the development of a large-scale RCT in community-dwelling older adults to compare the effectiveness of KOKU-Nut to an information booklet about healthy living. Recruitment rates will be assessed as cumulative recruitment against the target rate each month. Retention rates will be calculated as the number of participants who completed the study divided by the number of participants randomized. Engagement with the intervention will be based on self-reported use of KOKU-Nut at the end of the 12 weeks (every day, 3-4 times a week, 1-2 times a week, and once or twice a month). A questionnaire previously developed and used by the research team for the KOKU app will assess the acceptability of KOKU-Nut [13]. Intervention acceptability will also be assessed using the 11-item, 7-point, modified Treatment Evaluation Inventory [20]. Higher scores indicate higher acceptability, with a score of 44 indicating moderate acceptability. Interviews will explore the acceptability of the intervention and the perceived impact of taking part in the study. Interviews will also consider the participant’s experience of the recruitment process, the assessment tools used, and their experience and motivation for participating in the study (positive and negative).

**Figure 1:**
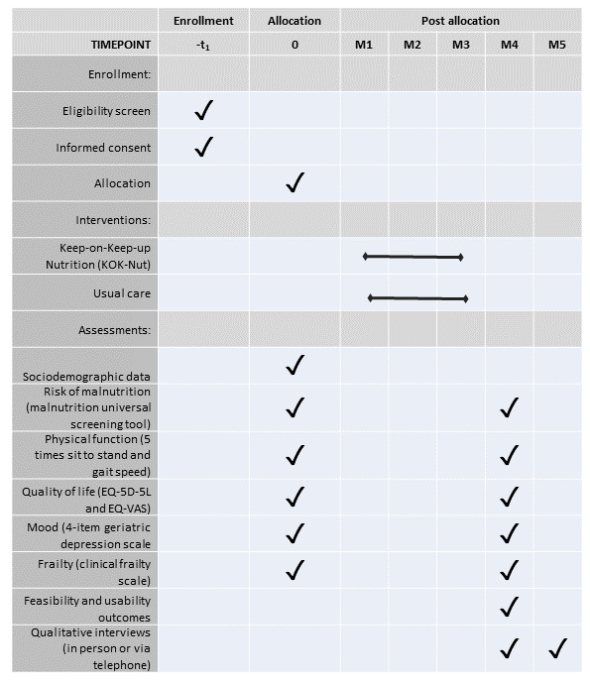
Schedule of enrollment, interventions, and assessments.

Secondary outcomes will consider the usability and safety of the intervention and the practicality of collecting and assessing the effectiveness of KOKU-Nut compared to an information booklet about healthy living. The perceived usability of KOKU-Nut will be assessed using the validated 10-item system usability scale. Responses are measured on a 5-point Likert scale ranging from 1 (strongly disagree) to 5 (strongly agree). A score >68 is considered above-average usability and >80 is considered high usability such that participants are likely to recommend the product to peers. The safety of the intervention will be based on the number of adverse events that occurred as a result of participation in the study. We will consider the practicality of collecting effectiveness data by documenting the amount and rationale for any missing data. Changes in means and SDs will be compared to consider the preliminary effectiveness of the intervention and to calculate sample sizes for a future trial. The study will be assessed against progression criteria based on Shanyinde et al [21] to determine progression to a powered RCT. Figure 1 shows the schedule of the intervention and assessment according to the SPIRIT statement.

### Sample Size

As this is a feasibility study that is not designed to detect statistically significant differences in measures, no formal power calculation has been conducted to determine the sample size. Lancaster et al [22] recommend a sample size of 36 participants for feasibility and pilot studies, so after accounting for potential dropout (at 20%), we aim to recruit 18 participants in each arm.

### Recruitment

Men and women aged 65 years and older living independently in the community will be recruited through purposive sampling via local age UK groups, assisted living facilities, and university networks. We plan to recruit a mixture of participants with and without access to a tablet computer to explore differences in the outcomes and the experiences between those with and without prior experience with tablets or iPads.

The study will be advertised using social media, posters, and through current collaborations with sheltered housing facilities, local charities, and community-based organizations across Greater Manchester. Staff at assisted living facilities, charities, and community organizations will act as gatekeepers and will offer potential participants flyers about the study and participant information sheets. Older adults who are interested in participating can agree to have their details passed over to the research team (CF) and will complete the consent to contact form. The research team will follow up with participants who are interested in joining the study and screen potential participants. Alternatively, potential participants may contact the research team directly following the study advertisement in posters and on social media to find out more information and check their eligibility. An existing research database will also be used where older adults have previously consented to be contacted about future research. Participants on the research database will be contacted directly by the research team with the participant information sheet and summary of the research according to their preferred choice (via email, telephone, or letter). The research team will then arrange to conduct an initial telephone screening over the phone to determine eligibility, provide further information about the study, and answer any questions.

**Figure 2:**
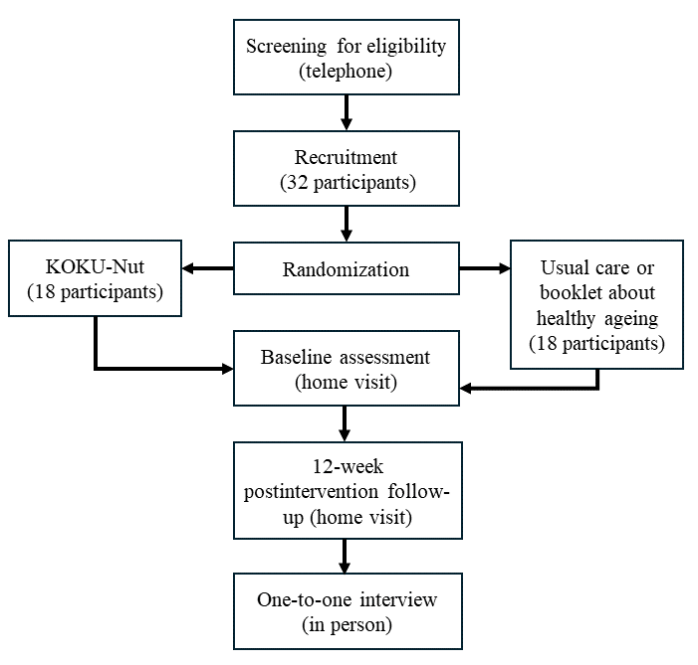
Flowchart of participants from screening to follow-up.

Recruitment for the qualitative interviews will be done purposefully, to recruit a demographically representative group of participants. To do this, the sample frame will be divided into a number of smaller groups, namely the treatment group, age, gender, and prior ownership of a tablet or iPad. Individuals will then be drawn at random from each of these groups. We also plan to contact any dropouts to understand their rationale for dropping out to optimize the retention of participants if continuing to a full RCT. Researchers will use the concept of information power [23] to determine the number of interviews, but we anticipate sufficient data will be obtained from interviewing 20% of participants recruited for the study. Figure 2 represents the overall flowchart of participants through the study.

Participants who meet the screening criteria and are interested in taking part will provide informed written consent (Multimedia Appendix 1) before being formally enrolled in the study. During the screening phone call, the researcher (CF) will arrange a date and time suitable for the participant to conduct the initial baseline assessment. Prior to completing baseline measures, participants will be randomized in a 1:1 ratio to receive the intervention (KOKU-Nut) or to the control group and receive usual care.

### Allocation

Randomization will be undertaken by a separate member of the research team using the Sealed Envelope randomization service [24], and participants will be stratified by age group (65-75 years and 76 years and older), gender, and prior experience of using a tablet or iPad. Given the nature of the intervention, participants and researchers will be unblinded.

### Data Collection Methods

#### Overview

All participants will receive in the post a baseline case report file to be completed before the baseline assessment to collect sociodemographic information including age, gender, ethnicity, marital status, occupation, use of digital technology, shopping, and cooking habits. Participants will also be asked to complete a 3-day food diary using Intake24 prior to the baseline visit [25]. This web-based dietary assessment tool is compliant with the general data protection regulations (2018), and participants will be sent a unique number that will enable the researcher to identify the digital dietary records. Data collected will be used to assess the consumption of different food groups to identify adherence to the Eatwell guide [14]. During the home visit, the researcher (CF) will collect baseline measurements to assess anthropometry, dietary intake, physical function, quality of life, mood, and frailty. All measurements are responsive to change, appropriate for use in this population, and easily assessed in a community setting.

At the end of the 12-week intervention period, all baseline measures will be repeated, and participants will complete a follow-up case report file to collect feasibility outcome data. The researcher will conduct one-to-one semistructured interviews with a selected group of participants from both the intervention and the control group. Interviews will be conducted in person or over the phone depending on the preference of the participant. Interviews will last approximately 60 minutes, will be audio-recorded, and then transcribed verbatim by the research team. Additional consent will be collected prior to conducting the interview. A topic guide developed for the study will be used flexibly to guide the interviews.

#### Anthropometry

A member of the research team will measure height to the nearest centimeter using a stadiometer (Harpenden pocket stadiometer Practical Metrology) and body weight to the nearest 0.1 kg (TANITA, Arlington Heights). BMI will be calculated and categorized according to standard cutoffs [26].

#### Dietary Assessment

A researcher (CF) who is also a registered nutritionist will use the malnutrition universal screening tool as recommended by the European Society for Clinical Nutrition and Metabolism to assess the risk of malnutrition [27].

#### Physical Function

Participants will be asked to complete the 5-times sit-to-stand physical function test. The 5-times sit-to-stand requires participants to rise from a chair 5 times as quickly as possible with arms folded across their chest and the researcher will record the time taken to complete the task. The researcher will then calculate lower limb muscle power using the validated equation developed by Kirk et al [28] and Alcazar et al [29]. Grip strength will be assessed on a Takei 5001 analog hand grip dynamometer (Takei Scientific Instruments Co, Ltd) 3 times on each hand with the best score used for analysis.

#### Quality of Life

The participants’ perceived state of health and quality of life will be assessed using the validated EQ-5D questionnaire in combination with the EQ-5D-5L [30]. The EQ-5D questionnaire comprises 5 questions assessing mobility, self-care, usual activities, pain or discomfort, and anxiety or depression to produce an overall score representing the participant’s health profile. The score will range from 1 (full health) to 0 (state of health equitable to death), with the option to have negative values representing a state of health considered to be worse than death [31]. The EQ-5D-5L is a visual scale ranging from 0 (worst health imaginable) to 100 (best health imaginable), where participants indicate how they perceive their current health status both on a number line and numerically [31].

#### Mood

Participants will complete the 4-item Geriatric depression scale as part of the baseline questionnaire, given that mood can affect motivation and may affect the use of KOKU-Nut.

#### Frailty

The researcher will assess frailty status based on the descriptions and pictographs included in the clinical frailty scale, which has been well validated and is frequently used in adults aged 65 years and older [32,33].

### Data Monitoring and Management

All digital and physical data will be stored securely at the University of Manchester and securely destroyed at the end of the data retention period as stated in the data management plan. Digital data will be stored on a backup drive on the university’s central server accessible by the research team. On enrollment to the study, all participants will be assigned a unique identification number with all subsequent data stored against this pseudonymized number. Identifiable information (such as name and contact details) will be stored separately and will only be linked to study-collected data through a recruitment log which will be password protected and file access will be limited to researchers working on the study. All printed documentation including consent forms and participants’ case files will be stored in a locked storage cabinet in a designated location at the University of Manchester. Interviews will be recorded on an encrypted Dictaphone. Audio recordings will be securely destroyed (digitally shredded) from the Dictaphone after being transcribed and validated by a member of the research team.

### Statistical Analysis

All quantitative data will be analyzed in STATA (version 15; StataCorp). The primary outcome will be calculated with regard to the indicators of feasibility to investigate engagement with the intervention, recruitment and retention rates, and acceptability of the intervention and study design. These will be summarized appropriately using descriptive statistics including percentages, mean with SDs, and ranges. Pre-post intervention changes in health measures will be analyzed in an exploratory fashion to gain preliminary insights into the number of valid versus missing data and to assess how outcomes are associated with group assignment and intervention engagement. Effect sizes will be determined using Cohen *d* to calculate sample sizes for a powered RCT.

Qualitative interview transcripts will be managed using NVivo software (version 12; QSR International Pty Ltd) and analyzed using an inductive approach with the 5 stages of framework analysis [34,35] to understand any barriers to the recruitment process, the suitability of the assessment measures, and the practicality of using the KOKU-Nut intervention. The first stage of framework analysis involves familiarization of the data from the transcripts and audio-recordings. The second stage involves classifying the data into codes and identifying a thematic framework from the key themes identified. The third stage involves systematically indexing the data using the framework identified. The fourth stage involves charting and synthesizing the data to categorize and capture key concepts and themes. The final stage involves the mapping and interpretation of the data [34]. All stages of the analysis process will be discussed and reviewed with at least 2 researchers (ES and SB) to ensure rigor [36].

### Ethical Considerations

Ethics approval was obtained from The University of Manchester Research Ethics Committee on August 18, 2023 (2023-17372-30569) to ensure that adequate safeguards are in place to protect the privacy of participants and to maintain the confidentiality of data. This study will be conducted in accordance with the UK Policy Framework for Health and Social Care Research and the Declaration of Helsinki guidelines. Prior to data collection, all the participants who agreed to participate in the study will sign a consent form, which includes a description of the study, its objectives, and participants’ involvement and rights (Multimedia Appendix 1). Participation in the study is entirely voluntary and participants can withdraw at any time. Participants will not receive financial compensation for their contribution to the study. Any deviations from the protocol will be put through the appropriate ethical amendment process. It is not anticipated that the study will be stopped prior to its intended end date. However, the study will be halted if safety issues arise regarding the intervention or resources to conduct the study are no longer available. Outcomes will be disseminated through conference presentations and publications in peer-reviewed journals in deidentified form. The findings will also be shared through presentations with digital inclusion teams and community stakeholders.

## Results

We anticipate recruitment will commence in March 2024 and will continue until enrollment is complete (N=36). The analysis is expected to be completed by November 2024, and the results will be published by January 2025.

## Discussion

### Principal Findings

The intervention (KOKU-Nut) involves educating users about how and why to make lifestyle changes, encouraging participation in regular and progressive strength and balance activities, and nudging older adults to improve their dietary intake. These features are underpinned by behavior change theories and have been co-designed with end users.

In comparison to many physical activity digital health tools such as Nymbl and Standing Tall, KOKU-Nut incorporates a nutritional component with a focus on dietary protein and fluid intake, given their low intakes among older adults [37,38] and their role in health [39,40]. The impact of diet is well established and can help older adults maintain health status and physical function [41]. Furthermore, it is estimated that one-quarter of Europeans older than the age of 65 years are at a high risk of malnutrition [42], and this can increase the risk of frailty, musculoskeletal conditions, and mortality [43-45]. In addition to the high prevalence of malnutrition, a recent James Lind Alliance priority setting partnership identified early intervention in vulnerable groups as a top priority [46].

There has been a rapid development in the number and quality of mobile health apps, and there is a growing acceptance among older adults. These tools have many benefits including that they can increase motivation through notifications and tracking of progress and provide personalized feedback to the user and increase engagement through gamification. However, there are still many challenges around introducing new digital health apps to this population. The usability of digital technology is important to ensure sustained use and to allow users to engage with the product for its intended purpose. Acceptability refers to the extent to which users consider the digital technology appropriate. Mobile apps are more likely to be usable and acceptable to older adults if they are codeveloped with them using principles of human-centered design [47,48]. KOKU-Nut was co-designed with end users and health care professionals, and we hope this will maximize the usability and usefulness of the interface and content.

This study will provide information on the feasibility and usability of the mobile health app KOKU-Nut. To our knowledge, this is the first digital health app in the United Kingdom designed to empower community-dwelling older adults to increase their activity levels and improve their diet and fluid intake so that they can stay active and healthy for longer.

### Study Limitations

This study has a number of limitations. We plan to recruit participants using purposive sampling from across Greater Manchester and thus this limits the generalizability of the results to the rest of the United Kingdom and internationally. However, this is a feasibility study that aims to inform a powered RCT that would then enable conclusions to be drawn. Furthermore, many of the study outcomes including dietary assessment and assessments of feasibility are self-reported and so prone to recall bias. Questionnaires will be checked for completeness and Intake24 appears to report similar data to intakes reported in interviewer-led dietary recall [49] and the gold standard assessment of energy intake using doubly labeled water [50].

### Conclusions

To our knowledge, this will be the first RCT to evaluate a digital health tool that nudges community-dwelling older adults living in the United Kingdom to improve their dietary intake and reduce the risk of malnutrition. The study aligns with guidelines developed by the Medical Research Council for developing a complex intervention by using qualitative and quantitative research to examine the barriers to intervention from the perspective of users and identify potential challenges around recruitment and retention [51].

## Appendix

Multimedia Appendix 1 Supplementary material to demonstrate SPIRIT checklist (S1), CONSORT checklist (S2) and Consent form (S3).

## Abbreviations

CONSORT: Consolidated Standards of Reporting Trials
KOKU: Keep-on-Keep-up
KOKU-Nut: Keep-on-Keep-up Nutrition
RCT: randomized controlled trial
SPIRIT: Standard Protocol Items: Recommendations for Interventional Trials

## Data Availability

We plan to write up the findings for publication, which will include an overview of the anonymized data. The data sets generated and analyzed during this study are not publicly available in accordance with the data management plan but are available from the corresponding author on reasonable request.
