# Supplementary material for: Digital Intervention (Keep-On-Keep-Up Nutrition) to Improve Nutrition in Older Adults: Protocol for a Feasibility Randomized Controlled Trial
Source: JMIR Res Protoc. 2024 Apr 30;13:e50922. doi: 10.2196/50922 (PMC11094602; doi:10.2196/50922)
Supplement: Multimedia Appendix 1 [file resprot_v13i1e50922_app1.docx]

**Supplementary information:**

**S1: SPIRIT Outcomes 2022 Checklist**

| **Section** | **Item No.** | **SPIRIT 2013 Item** | **SPIRIT Outcomes 2022 item** | **Location reported** |
| --- | --- | --- | --- | --- |
| Administrative information | | | | |
| Title | 1 | Descriptive title identifying the study design, population, interventions, and, if applicable, trial acronym | - | Page 1 |
| Trial registration | 2a | Trial identifier and registry name. If not yet registered, name of intended registry | - | Page 2 |
|  | 2b | All items from the World Health Organization Trial Registration Data Set | - | N/A |
| Protocol | 3 | Date and version identifier | - | Page 4 |
| Funding | 4 | Sources and types of financial, material, and other support | - | Page 12 |
| Roles and responsibilities | 5a | Names, affiliations, and roles of protocol contributors | - | Page 1, 12 |
|  | 5b | Name and contact information for the trial sponsor | - | N/A |
|  | 5c | Role of study sponsor and funders, if any, in study design; collection, management, analysis, and interpretation of data; writing of the report; and the decision to submit the report for publication, including whether they will have ultimate authority over any of these activities | - | N/A |
|  | 5d | Composition, roles, and responsibilities of the coordinating centre, steering committee, endpoint adjudication committee, data management team, and other individuals or groups overseeing the trial, if applicable (see Item 21a for data monitoring committee) | - | N/A |
| Introduction | | | | |
| Background and rationale | 6a | Description of research question and justification for undertaking the trial, including summary of relevant studies (published and unpublished) examining benefits and harms for each intervention | - | Page 3,4 |
|  | 6b | Explanation for choice of comparators | - | Page 5 |
| Objectives | 7 | Specific objectives or hypotheses | - | Page 4 |
| Trial design | 8 | Description of trial design including type of trial (eg. Parallel group, crossover, factorial, single group), allocation ratio, and framework (eg superiority, equivalence, non-inferiority, exploratory) | - | Page 4 |
| Methods: Participants, interventions, and outcomes | | | | |
| Study setting | 9 | Description of study settings (e.g. community clinic, academic hospital) and list of countries where data will be collected. Reference to where list of study sites can be obtained | - | Page 4 |
| Eligibility criteria | 10 | Inclusion and exclusion criteria for participants. If applicable, eligibility criteria for study centres and individuals who will perform the interventions (e.g. surgeons, psychotherapists) | - | Page 5 |
| Interventions | 11a | Interventions for each group with sufficient detail to allow replication, including how and when they will be administered (for specific guidance see TIDieR checklist and guide) | - | Page 5 |
|  | 11b | Criteria for discontinuing or modifying allocated interventions for a given trial participant (e.g. drug dose change in response to harms, participant request, or improving/ worsening disease) | - | N/A |
|  | 11c | Strategies to improve adherence to intervention protocols, and any procedures for monitoring adherence (e.g. drug tablet return, laboratory tests) | - | N/A |
|  | 11d | Relevant concomitant care and interventions that are permitted or prohibited during the trial | - | Page 5 |
| Outcomes | 12 | Primary, secondary and other outcomes, including the specific measurement variable (e.g. systolic blood pressure), analysis metric (e.g. change from baseline, final value, time to event), method of aggregation (e.g. median proportion), and time point for each outcome. Explanation of the clinical relevance of chosen efficacy and harm outcomes is strongly recommended | - | Page 5, 6 |
|  | 12.1 |  | Provide a rationale for the selection of the domain for the trial’s primary outcome | Page 5 |
|  | 12.2 |  | If the analysis metric for the primary outcome represents within-participant change, define and justify the minimal important change in individuals | N/A |
|  | 12.3 |  | If the outcome data collected are continuous but will be analysed as categorical (method of aggregation), specify the cutoff values to be used | N/A |
|  | 12.4 |  | If outcome assessments will be performed at several time points after randomization, state the time points that will be used for analysis | N/A |
|  | 12.5 |  | If a composite outcome is used, define all individual components of the composite outcome | Page 5, 6 |
| Participant timeline | 13 | Time schedule of enrolment, interventions (including any run-ins and washouts), assessments, and visits for participants. A schematic diagram is highly recommended (see Figure) | - | Page 6 (Figure 2) |
| Sample size | 14 | Estimated number of participants needed to achieve study objectives and how it was determined, including clinical and statistical assumptions supporting any sample size calculations | - | Page 7 |
|  | 14.1 |  | Define and justify the target difference between treatment groups (e.g. the minimal important difference) | Page 7 |
| Recruitment | 15 | Strategies for achieving adequate participant enrolment to reach target sample size | - | Page 7 |
| Methods: Assignment of intervention (for controlled trials) | | | | |
| Allocation: | | | | |
| Sequence generation | 16a | Method of generating the allocation sequence (e.g. computer-generated random numbers), and list of any factors for stratification. To reduce predictability of a random sequence, details of any planned restriction (e.g. blocking) should be provided in a separate document that is unavailable to those who enrol participants or assign interventions | - | Page 8 |
| Allocation concealment mechanism | 16b | Mechanism of implementing the allocation sequence (e.g. central telephone; sequentially numbered; opaque, sealed envelopes), describing any steps to conceal the sequence until interventions are assigned | - | Page 8 |
| Implementation | 16c | Who will generate the allocation sequence, who will enrol participants, and who will assign participants to interventions | - | Page 8 |
| Blinding (masking) | 17a | Who will be blinded after assignment to interventions (e.g. trial participants, care providers, outcome assessors, data analysts) and how | - | Page 8 |
|  | 17b | If blinded, circumstances under which un-blinding is permissible, and procedure for revealing a participant’s allocated intervention during the trial | - | N/A |
| Methods: Data collection, management and analysis | | | | |
| Data collection and methods | 18a | Plans for assessment and collection of outcome, baseline, and other trial data, including any related processes to promote data quality (e.g. duplicate measurements, training of assessors) and a description of study instruments (e.g. questionnaires, laboratory tests) along with their reliability and validity, if known. Reference to where data collection forms can be found, if not in the protocol | - | Page 8-10 |
|  | 18a.1 |  | Describe what is known about the responsiveness of the study instruments in a population similar to the study sample | Page 8, 9 |
|  | 18a.2 |  | Describe who will assess the outcome (e.g. nurse, parent) | Page 9,10 |
|  | 18b | Plans to promote participant retention and complete follow-up, including list of any outcome data to be collected for participants who discontinue or deviate from intervention protocols | - | Page 7 |
| Data management | 19 | Plans for data entry, coding, security, and storage, including any related processes to promote data quality (e.g. double data entry; range checks for data values). Reference to where details of data management procedures can be found, if not in the protocol | - | Page 10 |
| Statistical methods | 20a | Statistical methods for analysing primary and secondary outcomes. Reference to where other details of the statistical analysis plan can be found, if not in the protocol | - | Page 10 |
|  | 20a.1 |  | Describe any planned methods to account for multiplicity in the analysis or interpretation of the primary and secondary outcomes (e.g. co-primary outcomes, same outcome assessed at multiple time points, or subgroup analyses of an outcome) | Page 10 |
|  | 20b | Methods for any additional analyses (e.g. subgroup and adjusted analyses) | - | N/A |
|  | 20c | Definition of analysis population relating to protocol non-adherence (e.g. as randomised analysis), and any statistical methods to handle missing data (e.g. multiple imputation) | - | N/A |
| Methods: Monitoring | | | | |
| Data monitoring | 21a | Composition of data monitoring committee (DMC); summary of its role and reporting structure; statement of whether it is independent from the sponsor and competing interests; and reference to where further details about its charter can be found, if not in the protocol. Alternatively, an explanation of why a DMC is not needed | - | Page 10 |
|  | 21b | Description of any interim analyses and stopping guidelines, including who will have access to these interim results and make the final decision to terminate the trial | - | Page 11 |
| Harms | 22 | Plans for collecting, assessing, reporting, and managing solicited and spontaneously reported adverse events and other unintended effects of trial interventions or trial conduct | - | Page 11 |
| Auditing | 23 | Frequency and procedures for auditing trial conduct, if any, and whether the process will be independent from investigators and the sponsor | - | N/A |
| Ethics and dissemination | | | | |
| Research ethics approval | 24 | Plans for seeking research ethics committee/ institutional review board (REC/IRB) approval | - | Page 11 |
| Protocol amendments | 25 | Plans for communicating important protocol modifications (e.g. changes to eligibility criteria, outcomes, analyses) to relevant parties (e.g. investigators, REC/IRBs, trial participants, trial registries, journals, regulators) | - | Page 11 |
| Consent or asset | 26a | Who will obtain informed consent or assent from potential trial participants or authorised surrogates and how (see Item 32) | - | Page 11 |
|  | 26b | Additional consent provisions for collection and use of participant data and biological specimens in ancillary studies, if applicable | - | Page 10 |
| Confidentiality | 27 | How personal information about potential and enrolled participants will be collected, shared, and maintained in order to protect confidentiality before, during, and after the trial | - | Page 10 |
| Declaration of interests | 28 | Financial and other competing interests for principal investigators for the overall trial and each study site | - | Page 12 |
| Access to data | 29 | Statement of who will have access to the final trial dataset, and disclosure of contractual agreements that limit such access for investigators | - | Page 10 |
| Ancillary and post-trial care | 30 | Provisions, if any, for ancillary and post-trial care, and for compensation to those who suffer harm from trial participation | - | N/A |
| Dissemination policy | 31a | Plans for investigators and sponsor to communicate trial results to participants, healthcare professionals, the public, and other relevant groups (e.g. via publication, reporting in results databases, or other data sharing arrangements), including any publication restrictions | - | Page 11 |
|  | 31b | Authorship eligibility guidelines and any intended use of professional writers | - | N/A |
|  | 31c | Plans, if any, for granting public access to the full protocol, participant-level dataset, and statistical code | - | N/A |
| Appendices | | | | |
| Informed consent materials | 32 | Model consent form and other related documentation given to participants and authorised surrogates | - | Supplementary information |
| Biological specimens | 33 | Plans for collection, laboratory evaluation, and storage of biological specimens for genetic or molecular analysis in the current trial and for future use in ancillary studies, if applicable | - | N/A |

**S2: CONSORT checklist of information to include when reporting a pilot trial**

| **Section** | **Item No** | **Standard checklist item** | **Extension for pilot trials** | **Page No.** |
| --- | --- | --- | --- | --- |
| **Title and abstract** | | | | |
|  | 1a | Identification as a randomised trial in the title | Identification as a pilot or feasibility randomised trial in the title | Page 1 |
|  | 1b | Structured summary of trial design, methods, results, and conclusions (for specific guidance see CONSORT for abstracts) | Structured summary of pilot trial design, methods, results, and conclusions (for specific guidance see CONSORT abstract extension for pilot trials) | Page 2 |
|  | **Introduction** | | | |
| Background and objectives | 2a | Scientific background and explanation of rationale | Scientific background and explanation of rationale for future definitive trial, and reasons for randomised pilot trial | Page 3, 4 |
|  | 2b | Specific objectives or hypothesis | Specific objectives or research questions for pilot trial | Page 4 |
| **Methods** | | | | |
| Trial design | 3a | Description of trial design (such as parallel, factorial) including allocation ratio | Description of pilot trial design (such as parallel, factorial) including allocation ratio | Page 4 |
|  | 3b | Important changes to methods after trial commencement (such as eligibility criteria), with reasons | Important changes to methods after pilot trial commencement (such as eligibility criteria), with reasons | N/A |
| Participants | 4a | Eligibility criteria for participants |  | Page 4 |
|  | 4b | Settings and locations where the data were collected |  | Page 4 |
|  | 4c |  | How participants were identified and consented | Page 6, 7 |
| Interventions | 5 | The Interventions for each group with sufficient details to allow replication, including how and when they were actually administered |  | Page 5 |
| Outcomes | 6a | Completely defined pre-specified primary and secondary outcome measures, including how and when they were assessed | Completely defined pre-specified assessments or measurements to address each pilot trial objective specified in 2b, including how and when they were assessed | Page 5,6 |
|  | 6b | Any changes to trial outcomes after the trial commenced, with reasons | Any changes to pilot trial assessments or measurements after the pilot trial commenced, with reasons | N/A |
|  | 6c |  | If applicable, pre-specified criteria used to judge whether, or how, to proceed with future definitive trial | Page 6 |
| Sample size | 7a | How sample size was determined | Rationale for numbers in the pilot trial | Page 6 |
|  | 7b | When applicable, explanation of any interim analyses and stopping guidelines |  | N/A |
| Randomisation: | | | | |
| Sequence generation | 8a | Method used to generate the random allocation sequence |  | Page 8 |
|  | 8b | Type of randomisation: details of any restriction (such as blocking and bloc size) | Type of randomisation(s); details of any restriction (such as blocking and block size) | Page 8 |
| Allocation concealment mechanism | 9 | Mechanism used to implement the random allocation sequence (such as sequentially numbered containers), describing any steps taken to conceal the sequence until interventions were assigned |  | Page 8 |
| Implementation | 10 | Who generated the random allocation sequence, enrolled participants and assigned participants to interventions |  | Page 8 |
| Blinding | 11a | If done, who was blinded after assignment to interventions (eg participants, care providers, those assessing outcomes) and how |  | Page 8 |
|  | 11b | If relevant, description of the similarity of interventions |  | N/A |
| Analytical methods | 12a | Statistical methods used to compare groups for primary and secondary outcomes | Methods used to address each pilot trial objective whether qualitative or quantitative | Page 10 |
|  | 12b | Methods for additional analyses, such as subgroup analyses and adjusted analyses | Not applicable | N/A |
| **Results** | | | | |
| Participant flow (a diagram is strongly recommended) | 13a | For each group, the numbers of participants who were randomly assigned, received intended treatment, and were analysed for the primary outcome | For each group, the numbers of participants who were approached and/ or assessed for eligibility, randomly assigned, received intended treatment, and were assessed for each objective | N/A |
|  | 13b | For each group, losses and exclusions after randomisation, together with reasons |  | N/A |
| Recruitment | 14a | Dates defining the periods of recruitment and follow-up |  | N/A |
|  | 14b | Why the trial ended or was stopped | Why the pilot trial ended or was stopped | N/A |
| Baseline data | 15 | A table showing baseline demographic and clinical characteristics for each group |  | N/A |
| Numbers analysed | 16 | For each group, numbers of participants (denominator) included in each analysis and whether the analysis was by original assigned groups | For each objective, number of participants (denominator) included in each analysis. If relevant, these numbers should be by randomised group | N/A |
| Outcomes and estimation | 17a | For each primary and secondary outcome, results for each group, and the estimated effect size and its precision (such as 95% confidence interval) | For each objective, results including expression of uncertainty (such as 95% confidence interval) for any estimates. If relevant, these results should be by randomised group | N/A |
|  | 17b | For binary outcomes, presentation of both absolute and relative effect sizes is recommended | Not applicable | N/A |
| Ancillary analyses | 18 | Results of any other analyses performed, including subgroup analyses and adjusted analyses, distinguishing pre-specified from exploratory | Results of any other analyses performed that could be used to inform the future definitive trial | N/A |
| Harms | 19 | All important harms or unintended effects in each group (for specific guidance see CONSORT for harms) |  | N/A |
|  | 19a |  | If relevant, other important unintended consequences | N/A |
| **Discussion** | | | | |
| Limitations | 20 | Trial limitation, addressing sources of potential bias, imprecision, and, if relevant, multiplicity of analyses | Pilot trial limitations, addressing sources of potential bias and remaining uncertainty about feasibility | Page 11 |
| Generalisability | 21 | Generalisability (external validity, applicability) of the trial findings | Generalisability (applicability) of pilot trial methods and findings to future definitive trial and other studies | Page 11 |
| Interpretation | 22 | Interpretation consistent with results, balancing benefits and harms, and considering other relevant evidence | Interpretation consistent with pilot trial objectives and findings, balancing potential benefits and harms, and considering other relevant evidence | N/A |
|  | 22a |  | Implications for progression from pilot to future definitive trial, including any proposed amendments | Page 6 |
| **Other information** | | | | |
| Registration | 23 | Registration number and name of trial registry | Registration number for pilot trial and name of trial registry | Page 2 |
| Protocol | 24 | Where the full trial protocol can be accessed, if available | Where the pilot trial protocol can be accessed, if available | N/A |
| Funding | 25 | Sources of funding and other support (such as supply of drugs), role of funders |  | Page 12 |
|  | 26 |  | Ethical approval or approval by research review committee, confirmed with reference number | N/A |

**S3: Consent form**

**Exploring the feasibility of a digital service to improve nutrition and hydration in older adults: a mixed methods study**

If you are happy to participate, please complete and sign the consent form below.

|  | **Activities** | Initials |
| --- | --- | --- |
| 1 | I confirm that I have read the attached information sheet (**Version 2, Date 21/07/2023**) for the above study and have had the opportunity to consider the information and ask questions and had these answered satisfactorily. |  |
| 2 | I understand that my participation in the study is voluntary and that I am free to withdraw at any time without giving a reason and without detriment to myself. |  |
| 3 | I agree that any data collected may be included in anonymous form in publications/conference presentations. |  |
| 4 | I understand that data collected during the study may be looked at by individuals from The University of Manchester or regulatory authorities, where it is relevant to my taking part in this research. I give permission for these individuals to have access to my data. |  |
| 5 | I understand that there may be instances where during the course of the research information is revealed which means the researchers will be obliged to break confidentiality and this has been explained in more detail in the information sheet. |  |
| 6 | I am happy to provide details of my GP so that the research team can let them know about my participation in the study. I understand my decision to participate in this study will not affect my healthcare or treatment in any way. |  |
| 7 | I agree to take part in this study. |  |

**Please turn over**

**The following activities are optional; you may participate in the research without agreeing to the following:**

| 7 | I understand a subsample of participants will be invited to take part in an optional interview capturing their experience of the study. I consent to being contacted about this study. |  |
| --- | --- | --- |
| 8 | I agree that the researchers may contact me in future about other research projects |  |
| 9 | I agree that the researchers may retain my contact details in order to provide me with a summary of findings for this study. |  |

**Data Protection**

**The personal information we collect and use to conduct this research will be processed in accordance with UK data protection law as explained in the Participant Information Sheet and the Privacy Notice for Research Participants, which can be found online:** [**https://documents.manchester.ac.uk/display.aspx?DocID=37095**](https://documents.manchester.ac.uk/display.aspx?DocID=37095)

__________________ __________________

Name of Participant Signature Date

___________________ ___________________

Name of the person Signature Date

taking consent
